# Perfusion dephasing discriminated between coronary microvascular disease and multivessels coronary artery disease

**DOI:** 10.1186/1532-429X-16-S1-O60

**Published:** 2014-01-16

**Authors:** Amedeo Chiribiri, Shazia T Hussain, Adriana Villa, Nuno Bettencourt, Andreas Schuster, Geraint Morton, Gilion LTF Hautvast, Marcel Breeuwer, Eike Nagel

**Affiliations:** 1 Cardiovascular Imaging, King's College London, London, UK; 2 Cardiology, CentroHospitalardeGaia/Espinho, Porto, Portugal; 3 Cardiology and Pulmonology and Heart Research Center, Georg-August-University, Goettingen, Germany; 4 Philips Group Innovation - Healthcare Incubator, Eindhoven, Netherlands; 5 Philips Healthcare, Imaging Systems, Best, Netherlands; 6 Biomedical Engineering, Biomedical Image Analysis, Eindhoven University of Technology, Eindhoven, Netherlands

## Background

Non-invasive imaging methods cannot reliably differentiate reliably between coronary microvascular dysfunction (CMD) and multi-vessel coronary artery disease (including left main coronary artery disease; CAD) as both these conditions may result in diffuse myocardial ischemia, which on visual and quantitative analysis can have a similar appearance. Consequently, patients are subjected to invasive angiography, and coronary microvascular dysfunction is diagnosed after demonstrating normal coronary arteries in patients with myocardial ischemia. We hypothesized that the spatial and temporal distribution of the first-pass perfusion of the left ventricular myocardium is fundamentally different between these conditions. The aim of this study was to evaluate the spatial and temporal distribution of perfusion (perfusion dephasing analysis) to differentiate CMD and multivessel CAD.

## Methods

This study included a total of 52 patients. All subjects underwent adenosine-stress first-pass perfusion CMR. Patients with a visually positive perfusion cardiac magnetic resonance (CMR) and functional multivessel disease (fractional flow reserve (FFR) ≤0.8 in at least 2 vessels; n = 25) or a visually positive perfusion CMR and angiographically smooth coronary arteries (CMD Group; n = 27) were included. The datasets were analysed visually, quantified with Fermi deconvolution and assessed for perfusion dephasing (coefficient of variation of the time to peak of the myocardial signal intensity curve).

## Results

Visual and quantitative analysis allowed a reliable diagnosis of significant CAD, but could not distinguish patients with multivessel CAD from patients with CMD as the total extent and severity of ischemia was similar between the two groups. In patients with CAD, perfusion analysis showed significant dephasing proportional to the extent and severity of the epicardial stenoses (average perfusion dephasing in the multivessel CAD group 17% ± 8%). In contrast, in patients with CMD there was very little perfusion dephasing (7% ± 2%;p < 0.001). The best threshold to diagnose non-invasively coronary multivessel disease in presence of diffuse left ventricular ischemia was a perfusion dephasing > 10.1% (p < 0.001; area under the ROC curve = 0.96).

## Conclusions

In conclusion, perfusion-dephasing analysis, a novel method to measure temporal differences of myocardial perfusion, is highly accurate in distinguishing patients with functionally significant multivessel CAD from patients with CMD.

## Funding

The Centre of Excellence in Medical Engineering is funded by the Wellcome Trust and EPSRC under grant number WT 088641/Z/09/Z. The authors acknowledge financial support from the Department of Health via the National Institute for Health Research (NIHR) comprehensive Biomedical Research Centre award to Guy's and St Thomas' NHS Foundation Trust in partnership with King's College London.

